# Supplementary material for: Barriers to contraceptive use in humanitarian settings: Experiences of South Sudanese refugee women living in Adjumani district, Uganda; an exploratory qualitative study
Source: PLoS One. 2024 Mar 1;19(3):e0278731. doi: 10.1371/journal.pone.0278731 (PMC10906906; doi:10.1371/journal.pone.0278731)
Supplement: Attachment — Submitted filename: Rebuttal letter responding to reviewers.docx [file pone.0278731.s001.docx]

**Response to reviewers of PLOS ONE**

PONE-D-22-31975

Barriers to contraceptive use in humanitarian settings: Experiences of South Sudan refugee women living in Adjumani district, Uganda

**Submitted on April 28 2023.**

Following items included with submission of revised manuscript:

- Rebuttal letter responding to the points raised by the academic editor/reviewers
- Revised Manuscript with Track Changes
- Revised Manuscript without track changes
- Revised cover letter

**Journal requirements:**

When submitting your revision, we need you to address these additional requirements.

1. Please ensure that your manuscript meets PLOS ONE's style requirements, including those for file naming. The PLOS ONE style templates can be found at

<https://journals.plos.org/plosone/s/file?id=wjVg/PLOSOne_formatting_sample_main_body.pdf> and <https://journals.plos.org/plosone/s/file?id=ba62/PLOSOne_formatting_sample_title_authors_affiliations.pdf>.

**Response:** The authors looked at the PLOS ONE template and adjusted the manuscript according (see attached revised manuscript).

2. You indicated that you had ethical approval for your study. In your Methods section, please ensure you have also stated whether you obtained consent from parents or guardians of the minors included in the study or whether the research ethics committee or IRB specifically waived the need for their consent.

**Response:** This section has been revised as follows: Written consent was obtained from participants. For the emancipated minors (15-17 years) who were married and had children, we obtained consent from them. However, for the other minors, we obtained assent and consent from their parents/guardians. (see ethical statement section)

3. Please update your submission to use the PLOS LaTeX template. The template and more information on our requirements for LaTeX submissions can be found at <http://journals.plos.org/plosone/s/latex>.

**Response:** The PLOS LaTeX template has been used in the revised manuscript

4. We note that the grant information you provided in the ‘Funding Information’ and ‘Financial Disclosure’ sections do not match. When you resubmit, please ensure that you provide the correct grant numbers for the awards you received for your study in the ‘Funding Information’ section.

**Response:** This has been addressed**:** **Grant ID**: NICHE-UGA-288. The Nuffic grant through the International Institute of Social Studies Erasmus University, Rotterdam for Strengthening Education and Training Capacity in Sexual Reproductive Health & Rights in Uganda (SET-SRHR)

5. Thank you for stating the following in the Acknowledgments/Funding Section of your manuscript: “Grant ID: NICHE -UGA-288. This research was conducted with financial support from Nuffic grant through The International Institute of Social Studies Erasmus University, Rotterdam for Strengthening Education and Training Capacity in Sexual Reproductive Health & Rights in Uganda (SET-SRHR).”

We note that you have provided funding information that is not currently declared in your Funding Statement. However, funding information should not appear in the Acknowledgments section or other areas of your manuscript. We will only publish funding information present in the Funding Statement section of the online submission form.

Please remove any funding-related text from the manuscript and let us know how you would like to update your Funding Statement. Currently, your Funding Statement reads as follows:

“Funding was only availed to RA, the Principal Investigator through Nuffic grant through The International Institute of Social Studies Erasmus University, Rotterdam for Strengthening Education and Training Capacity in Sexual Reproductive Health & Rights in Uganda (SET-SRHR). The funders had to role in the study design, data collection and analysis, decision to publish or preparation of the manuscript.”

Please include your amended statements within your cover letter; we will change the online submission form on your behalf.

**Response:** The funding information has been declared in the funding statement as per the guidance. - Funding information has been removed from the Acknowledgment section and any other areas of our manuscript. The funding information has been included in the cover letter as guided. We acknowledge the support to change the online form on our behalf to include the amended funding statement as below.

“The Principal Investigator received funding from Grant ID: NICHE - UGA-288. Nuffic grant through The International Institute of Social Studies Erasmus University, Rotterdam for Strengthening Education and Training Capacity in Sexual Reproductive Health & Rights in Uganda (SET-SRHR).

The funders had no role in the study design, data collection and analysis and did not make the decision to publish or preparation of this manuscript”.

6. We note that you have indicated that data from this study are available upon request. PLOS only allows data to be available upon request if there are legal or ethical restrictions on sharing data publicly. For more information on unacceptable data access restrictions, please see <http://journals.plos.org/plosone/s/data-availability#loc-unacceptable-data-access-restrictions>.

In your revised cover letter, please address the following prompts:

a) If there are ethical or legal restrictions on sharing a de-identified data set, please explain them in detail (e.g., data contain potentially sensitive information, data are owned by a third-party organization, etc.) and who has imposed them (e.g., an ethics committee). Please also provide contact information for a data access committee, ethics committee, or other institutional body to which data requests may be sent.

**Response:** This has been addressed in the cover letter as follows: There is no legal restriction for sharing de-identified data set. However, this is part of the bigger study and data can only be shared upon successful submission of the entire project. For now, we have relevant data included within the manuscript and supporting information.

b) If there are no restrictions, please upload the minimal anonymized data set necessary to replicate your study findings as either Supporting Information files or to a stable, public repository and provide us with the relevant URLs, DOIs, or accession numbers. For a list of acceptable repositories, please see <http://journals.plos.org/plosone/s/data-availability#loc-recommended-repositories>.

We will update your Data Availability statement on your behalf to reflect the information you provide.

**Response:** We have not uploaded the de-identified data set. We acknowledge your support to update for us the Data Availability statement on our behalf to reflect the information provided as above.

**[Note: HTML markup is below**. Please do not edit.]
Reviewers' comments: Reviewer's Responses to Questions

**Comments to the Author**
1. Is the manuscript technically sound, and do the data support the conclusions?
The manuscript must describe a technically sound piece of scientific research with data that supports the conclusions. Experiments must have been conducted rigorously, with appropriate controls, replication, and sample sizes. The conclusions must be drawn appropriately based on the data presented.

Reviewer #1: Yes

Reviewer #2: No

2. Has the statistical analysis been performed appropriately and rigorously?

Reviewer #1: N/A

Reviewer #2: N/A

3. Have the authors made all data underlying the findings in their manuscript fully available?

The [PLOS Data policy](http://www.plosone.org/static/policies.action#sharing) requires authors to make all data underlying the findings described in their manuscript fully available without restriction, with rare exception (please refer to the Data Availability Statement in the manuscript PDF file). The data should be provided as part of the manuscript or its supporting information, or deposited to a public repository. For example, in addition to summary statistics, the data points behind means, medians and variance measures should be available. If there are restrictions on publicly sharing data—e.g. participant privacy or use of data from a third party—those must be specified.

Reviewer #1: Yes

Reviewer #2: Yes

4. Is the manuscript presented in an intelligible fashion and written in standard English?

PLOS ONE does not copyedit accepted manuscripts, so the language in submitted articles must be clear, correct, and unambiguous. Any typographical or grammatical errors should be corrected at revision, so please note any specific errors here.

Reviewer #1: Yes

Reviewer #2: No

5. Review Comments to the Author

Please use the space provided to explain your answers to the questions above. You may also include additional comments for the author, including concerns about dual publication, research ethics, or publication ethics. (Please upload your review as an attachment if it exceeds 20,000 characters)

**Reviewer #1**: The paper studied a unique and often disadvantage population, thus will make the work attract readership. But there are a few technical issues the authors need to address.
1. The authors did not present any responses from the age group 15-19, adolescents experiences of barriers surely differs from older women. It will add value to filter responses from such age group and reflect such.

**Response:** Thank you for this concern, indeed the adolescents’ experiences of barriers to contraceptive use are crucial. This has been included in the result section with a quote and also in the discussion sections of the revised manuscript. For example, *"...Some parents say that adolescent girls may have babies with missing body parts. So, any abnormality is associated with use of FP. Even those who don’t produce after marriage are labelled as having used FP at early age. FP use among young people is very difficult in the settlements.* (IDI_ Female 15-19_Nyumanzi Settlement).

2. The authors choice and use of word "the research participant" made the findings/result section boring. This word reflect in every theme. Kindly revise.

**Response:** Yes, the monotony was noted and has been revised to avoid boring presentation of results section. Alternative words have been selected and used as in the revised manuscript.

3. Discussion: The discussion section needs to be revised to allow for synergy in the barriers identified. Also the authors is advised to sieve all recommendations listed with this section to feed into the "conclusion and recommendation" section.

**Response:** The discussion section has been revised as per the revised manuscript. Some recommendations listed in discussion section have also been filtered and taken to conclusion and recommendation section.

4. The authors should clearly cone down on how much of a barrier are the intra personal level factors, inter personal level and community level. Are there specific demographics to be focused for programming. If data is available, how do the Refugee host community reflect their country of origin.

**Response:** This has been addressed in line with the ecological model which clearly demonstrated the different factors at different levels. (see Fig 1. The Ecological model)

The specific demographics to be focused for programming are age and economic status. The adolescents whose access is affected by the cultural norms of early marriage, policy frameworks and the need for many children as they are married off at young age. Due to poverty, women remain dependent on men for their socio-economic support.

Given the available data, the Refugee host community are privileged with various sources of information and the government programs that promote family planning. Thus, have better options in their own country. However, because of the above factors, they still face barriers at different levels that affect their decision to use contraceptives.

5. The study did not talk about availability of methods/ providers (Access to FP services with the host communities).

**Answer**: Data on the available methods of family planning have not been considered in the revised manuscript. However, the most preferred method among the refugee community was condoms, whose access was easy within the settlements through health facilities and the community health workers. Other methods were also available like injectables and pills. Although, the women did not mention any significant barrier related to method availability, but the issue of choice was eminent and has been cited in the result section including other cultural norms that affected the way decisions are made to access contraception.

**Reviewer #2:** This manuscript explores barriers to contraceptive use in a humanitarian setting, specifically South Sudan refugees living in Uganda. The article makes a great contribution to our understanding of the barriers faced by women living in a humanitarian setting in accessing sexual and reproductive health services. However, several issues need to be addressed to improve the paper.

**Overall comments**
•To ensure that all necessary components of the study are reported, use the COREQ checklist

**Response:** Yes, the consolidated criteria for reporting qualitative research (COREQ checklist) (Tong Alison, 2007) has been used to report the relevant components of the study as in the revised manuscript.

•The authors indicated the study was guided by the socioecological model; however, the analysis and results sections seem not to have been guided by the model as the key findings/themes are not mapped according to the various ecological levels.

**Response:** The analysis and result sections of this study has been revised and presented in line with the socioecological model. For example in the analysis, the participants involved in this study were women between the ages of 15-49 years. A total of 32 participants were involved in FGDs and fourteen women reached with IDIs). To avoid repetition, we have sign posted readers to the conceptual framework which shows the results. Some factors were found to be at more than one level.

The result section has been guided by the model as follows:
 1) At intrapersonal level, the factors that affected contraceptive use were, demographic and social factors. These included age, education, economic status/occupation, religion, limited knowledge and lack of partner support due to negative attitudes on contraception.

2) At the interpersonal level, the factors were due to significant others which included Husbands/men’s negative attitudes, service providers, community/clan leaders antagonism on contraception**,** gender dynamics and reprisal for use of contraception.
3) At community level, the factors included cultural norms, socio economic factors, antagonism by Religious leaders, misconceptions, Gender dynamics.

Comments
•Several statements and data have been made without being referenced. Lines 62-64, 66-68, 69-70, 79-81, and 104-110 illustrate statements that should have been cited.

Review the entire document to ensure all statements and data are correctly referenced.
•Review the statistics given from lines 104-108. No need to include the n-values.

**Response:** Yes, the authors have taken note and made revisions as in the revised manuscript. Proper citation has also been done.

•The introduction section is quite lengthy and does not flow. Restructure the content to ensure consistency and brevity in the flow of ideas.

**Response:** The introduction section has been revised accordingly.

•Revise line 176-research assistance as opposed to research assistants.

**Response:** The authors took note of the error and changed to research assistants in the revised manuscript

•Repetition of the content listed under data collection and piloting the interview schedule. Did the study conduct both a pilot study and pretesting, if yes, what were their objectives?

**Response:** Only the pre-test was conducted. The objective was to check clarity of questions and the understanding by participants. Secondly, it was to ensure familiarity of the tool by the research assistants.

•There is inadequate information about the study participants. How were the participants recruited? What were the inclusion/exclusion criteria? Who participated in IDIs versus FGDs? A table will be helpful to show the participants who were interviewed. Apart from age, were there any socio-demographic characteristics of the women collected to better understand who was interviewed?

**Response:** the authors have revised this section and included a table of study participants, brief on inclusion/exclusion criteria and other socio-demographic characteristics of the participants as per the revised manuscript.

•In the results section, the first quote used in the subtheme (Joint decision…) doesn’t fully fit. It illustrates contraceptive decisions being driven by men, and not jointly by the couple.

**Response:** Although the contraceptive decision is driven by men, the end result is that they jointly agree to have many children and thus avoid contraceptive use. This is different from some women who do not agree to the suggestions by the partner and still go ahead to use contraceptives in hiding. Therefore, the authors have not changed the quote basing on that argument.

•Need to review when to use family planning and contraceptives.

**Response**: The authors have taken note. Family planning is used interchangeably with contraceptives given the circumstance and when referring to methods

•Line 362-Revise the title from discussions to discussion.
•The discussion section fails to compare and contrast previous studies done in similar settings.

**Response**: The authors have taken note of the comments on the discussion section and considered comparing and contrasting previous studies.

•Paraphrasing the results without referencing other studies: Are these findings unique to
•Referencing and citation are poorly done across the document. Review the reference style both in the text and the reference list to ensure it conforms to the journal’s guidelines.

**Response**: We have addressed the concerns as per the revised manuscript.

6. PLOS authors have the option to publish the peer review history of their article ([what does this mean?](https://journals.plos.org/plosone/s/editorial-and-peer-review-process#loc-peer-review-history)). If published, this will include your full peer review and any attached files.

If you choose “no”, your identity will remain anonymous but your review may still be made public.

**Do you want your identity to be public for this peer review?** For information about this choice, including consent withdrawal, please see our [Privacy Policy](https://www.plos.org/privacy-policy).

Reviewer #1: No

Reviewer #2: No

 [NOTE: If reviewer comments were submitted as an attachment file, they will be attached to this email and accessible via the submission site. Please log into your account, locate the manuscript record, and check for the action link "View Attachments". If this link does not appear, there are no attachment files.]
While revising your submission, please upload your figure files to the Preflight Analysis and Conversion Engine (PACE) digital diagnostic tool, <https://pacev2.apexcovantage.com/>. PACE helps ensure that figures meet PLOS requirements. To use PACE, you must first register as a user. Registration is free. Then, login and navigate to the UPLOAD tab, where you will find detailed instructions on how to use the tool. If you encounter any issues or have any questions when using PACE, please email PLOS at [figures@plos.org](mailto:figures@plos.org). Please note that Supporting Information files do not need this step.
