# Supplementary material for: Barriers to contraceptive use in humanitarian settings: Experiences of South Sudanese refugee women living in Adjumani district, Uganda; an exploratory qualitative study
Source: PLoS One. 2024 Mar 1;19(3):e0278731. doi: 10.1371/journal.pone.0278731 (PMC10906906; doi:10.1371/journal.pone.0278731)
Supplement: Attachment — Submitted filename: Response to Reviewers- PLOS ONE.rtf [file pone.0278731.s002.rtf]

POINT BY POINT RESPONSE TO REVIEWERS OF PLOS ONE

Title: Barriers to contraceptive use in humanitarian setting: Experiences of South Sudan refugee women living in Adjumani district, Uganda; An exploratory qualitative study

PONE-D-22-31975

Resubmitted on 26^th^ September, 2023. 

Following items are included with submission of revised manuscript:
•	Rebuttal letter responding to the points raised by the academic editor and reviewers 
•	Revised Manuscript with Track Changes
•	Revised Manuscript without track changes
Response to reviewers of PLOS ONE
Authors Response: Thank you so much for reviewing our manuscript. We appreciate all the comments that have been provided to us by the reviewers. We have responded to all the comments and references are made to the copy of manuscript with track changes. We have also taken note of the reference. We have also addressed the comments on point by point basis as below.

If you would like to make changes to your financial disclosure, please include your updated statement in your cover letter. Guidelines for resubmitting your figure files are available below the reviewer comments at the end of this letter.

If applicable, we recommend that you deposit your laboratory protocols in protocols.io to enhance the reproducibility of your results. Protocols.io assigns your protocol its own identifier (DOI) so that it can be cited independently in the future. For instructions see: https://journals.plos.org/plosone/s/submission-guidelines#loc-laboratory-protocols. Additionally, PLOS ONE offers an option for publishing peer-reviewed Lab Protocol articles, which describe protocols hosted on protocols.io. Read more information on sharing protocols at https://plos.org/protocols?utm_medium=editorial-email&utm_source=authorletters&utm_campaign=protocols.
We look forward to receiving your revised manuscript.

Response: It is not possible to deposit the protocol of this study in the repository yet, because this is the initial part of the entire project for the award of Doctoral degree. It is a requirement to publish atleast 3 papers and I may share for future reproducibility of the results at an appropriate time when the entire study has been concluded. 

Journal Requirements:

Please review your reference list to ensure that it is complete and correct. If you have cited papers that have been retracted, please include the rationale for doing so in the manuscript text, or remove these references and replace them with relevant current references. Any changes to the reference list should be mentioned in the rebuttal letter that accompanies your revised manuscript. If you need to cite a retracted article, indicate the article's retracted status in the References list and also include a citation and full reference for the retraction notice.
Response: Thank you so much for your comments. We are happy to note that we have added more new references to this manuscript. Refer to the Reference numbers 5,6, 8,10, 11, 14, 15, 16, 17, 22, 23, 29, 32,33, 34 and 35.
Review Comments to the Author

Please use the space provided to explain your answers to the questions above. You may also include additional comments for the author, including concerns about dual publication, research ethics, or publication ethics. (Please upload your review as an attachment if it exceeds 20,000 characters)
Reviewer #3: Whilst this manuscript addresses an important issue, and presents interesting data, overall, there are many typos and grammatical errors, which distract from the content. The entire manuscript would benefit from a close copy edit. See below for specific comments on errors picked up. I have also made a few general comments for the authors to consider, as well as suggestions for further reading which may help to enhance the discussion.
Comment: Title on Cover page has spelling error
Response: We have taken note, and corrected the spelling error in the manuscript 
Comment: Line 35: contraceptive use is lifesaving? or contraceptives can be lifesaving?
Response: This has been revised as contraceptive can be lifesaving in the manuscript- line 35.
Comment: Lines 37-38: I would avoid the term 'significant other' in academic writing – what does it really mean? A more objective precise term would be sexual/ romantic partner.
Response: Use of significant other in this study meant the family members including husbands, Father and mothers in law including other siblings and in laws who have stake in the woman's life. Therefore, this has been replaced with family member. See line 38. 
Comment: Line 46: “women of similar categories” – do you mean participants were as homogenous as possible, with similar characteristics in terms of age, gender e.t.c?
response: The participants were women of reproductive age ((15-49years). They were not homogeneous because some were adolescents, young women and others were mature adults. some were users and others not users of contraception. The only unifying thing is that they were all refugees from South Sudan and women of reproductive age. See line 46.
Comment: Line 70: Could remove the 'And' at the start of the sentence.
Response: The word “And” has been removed and sentenced reconstructed as indicated in line 70.
Comment: Line 75: typo: pillar
Response: The right spelling of pillar has been considered. See line 75
Comment: Line 88: barriers plural?
Response: The word barrier is singular and has been corrected in the manuscript, line 88
Comment: Line 90: Could remove the 'And' at the start of the sentence – just star the sentence with “yet”
Response: The word “And” has been removed, see line 91
Comment: Line 100: how are they defined as being 'vulnerable'?
Response: Women and girls are vulnerable because they need special care, support, and protection during humanitarian crisis. They have limited access to SRHR services including contraception that exposes them vulnerability. See lines 103-108
Comment: Line 111: rather than 'beating' preferable to use a term like 'intimate partner violence.'
Response: The word beating has been replaced with intimate partner violence in the manuscript, line 118
Comment: Line 124: Rather than writing “Mcleroy added; I quote” – it is preferable in a manuscript to paraphrase the quote and reference it, rather than writing in the first person.
Response: This has been corrected and proper quote with right refencing done. See lines 131-133
Comment: Line 129: describes 
Response: Correction has been done and word is describe. See line 136
Comment: Line 143: “a purposive sampling approach”
Response: Thank you, we have taken note and the sentence now reads; A purposive sampling approach was used to select participants for FGDs and IDIs. See line 149.
Comment: Line 148: poorly written - revise the sentence
Response: Sentence revised as in the manuscript line. See line 151-153
Comment: Line 162: were translated
Response: The sentence has been revised as in the manuscript, line 174
Comment: Line 178: “the next step”
Response: We have taken note and the word “the” has been added in line 187
Comment: Line 234: “the influence of men”
Response: The word “the” has been added in line 244
Comment: Line 236: “their husbands' involvement”
Response: The word “their” has been added in the sentence, line 246
Comment:  Line 237” check grammar and tenses of verbs
Response: This has been addressed, lines 246-247
Comment: Lines 253-254: words missing? Revise sentences
Response: The sentence has been revised as in lines 261-265.
Comment: Line 326: “institutional” does not need to be capitalized
Response: This has been considered and word “institution” corrected, line 398. 
Comment: Lines 333-335: this information about the Sexual and Reproductive Health policy guidelines should be in the discussion section, not the findings
Response: This section has been taken to the discussion. See lines 597-600
Comment: Line 343: grammar errors
Response: The sentence has been revised accordingly
Comment: Line 344: close the brackets
Response: Bracket closed as (LARCS), see line 413
Comment: Line 345: revise sentence
Response: The sentence has been revised as in the manuscript lines 419-422
Comment: Line 351: word missing – revise line 424 - 426
Response: Sentence revised as in the manuscript, lines 347-349
Comment: Line 353: sentence incomplete
Response: This has been revised as in lines 443-444
Comment: Line 354: “take blood samples” line 423
Response: Sentence revised accordingly as in line 361 of the manuscript
Comment: Line 384: “campaign against” – not de-campaign
Response: The right statement campaign against has been considered. See line 368
Comment: Line 413: grammar errors – revise
Response: The statement has been revised. See lines 393-396
Comment: Line 416: revise – remove reference to the researcher – rather refer to the authors, or the study
Response: We taken note of the comment on reference to the researcher and revised the statement as in line 425. 
Comment: Line 436: decision making? 
Response: Thank you, we have included the word “making” in lines 439-479.
Comment: Line 455: grammar errors
Response: This has been revised and the sentence now reads as in lines 480-482 & 488
Comment: In the discussion, the authors refer to “empowerment”. Whilst the concept of “empowerment” has been central to efforts to address gendered inequalities and power imbalances, thereby accelerating development and improving the health and well-being of women and girls. Empowerment is framed as a process of enhancing an individual's capacity to exercise choice, make decisions, and critically, the ability to act on those decisions, and achieving their choice. Interventions that aim to “empower” women and girls are designed to increase self-efficacy and agency, and therefore improve sexual and reproductive decision-making and related health outcomes – including the decision to use contraceptives or not. As this study demonstrates, since socio-cultural gendered norms and expectations are a key obstacle to women's exercise of agency in choosing to use contraceptives or not – as pervasive gendered sexual norms tend to give men greater decision-making power in sexual and reproductive matters - it is critical that interventions and programmes that aim to improve women's access to contraceptives address these gendered power inequalities by challenging gender norms and hegemonic masculinities of dominance. However, it also important to consider how programmes that aim to “empower” women and girls are cognisant of the danger of men feeling emasculated or threatened by women's empowerment, and the possibility for gender based violence and intimate partner violence as a reaction to this increased empowerment. Efforts to implement empowerment programming aimed at increasing women's self-efficacy for contraceptive use must consider the way in which dyadic level sexual scripts and relationship dynamics, including unequal power, are likely to constrain women's enactment of their decision making. Given the influence of socio-cultural level norms and hegemonic masculinities in sub-Saharan Africa, further attention needs to be paid to community level intervention approaches that aim to create conducive environments that enable women to exert their own agency and decision making in contraceptive use by shifting problematic gender norms and restrictive masculinities, and addressing gender inequalities that exist at the structural level.
Prior research from the sub-Saharan African setting has indicated that men feel anxiety around the concept of female-controlled methods of contraception due to the belief that men should control fertility. There is a need to consider most appropriate ways of giving women more agency in their sexual and reproductive health and choices, as this may be perceived by men as a challenge to their control.

Suggested references :
- Karp C, Wood SN, Galadanci H, Sebina Kibira SP, Makumbi F, Omoluabi E, Shiferaw S, Seme A, Tsui A, Moreau C. (2020). 'I am the master key that opens and locks': Presentation and application of a conceptual framework for women's and girls' empowerment in reproductive health. Soc Sci Med, 258. https://doi. org/10.1016/j.socscimed.2020.113086.
- Robinson JL, Narasimhan M, Amin A, Morse S, Beres LK, Yeh PT, et al. Interventions to address unequal gender and power relations and improve self-efficacy and empowerment for sexual and reproductive health decision-making for women living with HIV: a systematic review. PLoS ONE. 2017;12(8):e0180699. https:// doi.org/10.1371/journal.pone.0180699.
- Duby, Z., Bergh, K., Jonas, K. et al. “Men Rule… this is the Normal Thing. We Normalise it and it's Wrong”: Gendered Power in Decision-Making Around Sex and Condom Use in Heterosexual Relationships Amongst Adolescents and Young People in South Africa. AIDS Behav (2022). https://doi.org/10.1007/s10461-022-03935-8
- Pool, R., G. Hart, G. Green, S. Harrison, S. Nyanzi, and J. Whitworth. 2000. “Men's Attitudes to Condoms and Female Controlled Means of Protection against HIV and STDs in South-Western Uganda.” Culture, Health & Sexuality 2 (2): 197–211.
Response: Thank you so much for the well though literature. We have taken note and have incorporated the findings in this study. The suggested reference has also been considered appropriately in the manuscript.
Reviewer #4: I see the authors have tried to address the previous reviewers comments. However, the use of COREQ is not clearly adhered to... there is no description of who conducted the interviews (gender, prior experience or relationship with the participants, and age cat of ther interveiwers...?). This information will strengthen the methodology followed in conducting and reporting this study...
the table of demographics is also not consistent and unclear why there is a column for % in the categories but none for the total column...? Also, there is use of decimal points sometimes and other times not...?

Otherwise, the paper is an important paper!
Response: we have reviewed the methodology and incorporated the COREQ by including the details of the interviewers in the methodology section, line 167- 172. We have also reviewed the table of demographic characteristics and attached the revised copy.
7. PLOS authors have the option to publish the peer review history of their article (what does this mean?). If published, this will include your full peer review and any attached files.

If you choose “no”, your identity will remain anonymous but your review may still be made public.
Do you want your identity to be public for this peer review? For information about this choice, including consent withdrawal, please see our Privacy Policy.
Reviewer #3: No
Reviewer #4: Yes: Kim Jonas, PhD
[NOTE: If reviewer comments were submitted as an attachment file, they will be attached to this email and accessible via the submission site. Please log into your account, locate the manuscript record, and check for the action link "View Attachments". If this link does not appear, there are no attachment files.]

While revising your submission, please upload your figure files to the Preflight Analysis and Conversion Engine (PACE) digital diagnostic tool, https://pacev2.apexcovantage.com/. PACE helps ensure that figures meet PLOS requirements. To use PACE, you must first register as a user. Registration is free. Then, login and navigate to the UPLOAD tab, where you will find detailed instructions on how to use the tool. If you encounter any issues or have any questions when using PACE, please email PLOS at figures@plos.org. Please note that Supporting Information files do not need this step.

In compliance with data protection regulations, you may request that we remove your personal registration details at any time. (Remove my information/details). Please contact the publication office if you have any questions.
