# Supplementary material for: Barriers to contraceptive use in humanitarian settings: Experiences of South Sudanese refugee women living in Adjumani district, Uganda; an exploratory qualitative study
Source: PLoS One. 2024 Mar 1;19(3):e0278731. doi: 10.1371/journal.pone.0278731 (PMC10906906; doi:10.1371/journal.pone.0278731)
Supplement: Attachment — Submitted filename: Response to Reviewers- PLOS ONE.rtf [file pone.0278731.s003.rtf]

POINT BY POINT RESPONSE TO REVIEWERS OF PLOS ONE

PONE-D-22-31975R2
Barriers to contraceptive use in humanitarian setting: Experiences of South Sudanese refugee women living in Adjumani district, Uganda; An exploratory qualitative study
PLOS ONE


Dear Dr. Achola,

Thank you for submitting your manuscript to PLOS ONE. After careful consideration, we feel that it has merit but does not fully meet PLOS ONE's publication criteria as it currently stands. Therefore, we invite you to submit a revised version of the manuscript that addresses the points raised during the review process.

Please submit your revised manuscript by Dec 21 2023 11:59PM. If you will need more time than this to complete your revisions, please reply to this message or contact the journal office at plosone@plos.org. When you're ready to submit your revision, log on to https://www.editorialmanager.com/pone/ and select the 'Submissions Needing Revision' folder to locate your manuscript file.

Please include the following items when submitting your revised manuscript:
•	A rebuttal letter that responds to each point raised by the academic editor and reviewer(s). You should upload this letter as a separate file labeled 'Response to Reviewers'.
•	A marked-up copy of your manuscript that highlights changes made to the original version. You should upload this as a separate file labeled 'Revised Manuscript with Track Changes'.
•	An unmarked version of your revised paper without tracked changes. You should upload this as a separate file labeled 'Manuscript'. 
If you would like to make changes to your financial disclosure, please include your updated statement in your cover letter. Guidelines for resubmitting your figure files are available below the reviewer comments at the end of this letter.

If applicable, we recommend that you deposit your laboratory protocols in protocols.io to enhance the reproducibility of your results. Protocols.io assigns your protocol its own identifier (DOI) so that it can be cited independently in the future. For instructions see: https://journals.plos.org/plosone/s/submission-guidelines#loc-laboratory-protocols. Additionally, PLOS ONE offers an option for publishing peer-reviewed Lab Protocol articles, which describe protocols hosted on protocols.io. Read more information on sharing protocols at https://plos.org/protocols?utm_medium=editorial-email&utm_source=authorletters&utm_campaign=protocols.

We look forward to receiving your revised manuscript.
Kind regards,
Adetayo Olorunlana, Ph.D.
Academic Editor
PLOS ONE
Journal Requirements:

Please review your reference list to ensure that it is complete and correct. If you have cited papers that have been retracted, please include the rationale for doing so in the manuscript text, or remove these references and replace them with relevant current references. Any changes to the reference list should be mentioned in the rebuttal letter that accompanies your revised manuscript. If you need to cite a retracted article, indicate the article's retracted status in the References list and also include a citation and full reference for the retraction notice.

[Note: HTML markup is below. Please do not edit.]

Reviewers' comments:

Reviewer's Responses to Questions
Comments to the Author

1. If the authors have adequately addressed your comments raised in a previous round of review and you feel that this manuscript is now acceptable for publication, you may indicate that here to bypass the “Comments to the Author” section, enter your conflict of interest statement in the “Confidential to Editor” section, and submit your "Accept" recommendation.
Reviewer #3: (No Response)

2. Is the manuscript technically sound, and do the data support the conclusions?

The manuscript must describe a technically sound piece of scientific research with data that supports the conclusions. Experiments must have been conducted rigorously, with appropriate controls, replication, and sample sizes. The conclusions must be drawn appropriately based on the data presented.
Reviewer #3: Yes

3. Has the statistical analysis been performed appropriately and rigorously?
Reviewer #3: N/A

4. Have the authors made all data underlying the findings in their manuscript fully available?

The PLOS Data policy requires authors to make all data underlying the findings described in their manuscript fully available without restriction, with rare exception (please refer to the Data Availability Statement in the manuscript PDF file). The data should be provided as part of the manuscript or its supporting information, or deposited to a public repository. For example, in addition to summary statistics, the data points behind means, medians and variance measures should be available. If there are restrictions on publicly sharing data—e.g. participant privacy or use of data from a third party—those must be specified.
Reviewer #3: Yes

5. Is the manuscript presented in an intelligible fashion and written in standard English?

PLOS ONE does not copyedit accepted manuscripts, so the language in submitted articles must be clear, correct, and unambiguous. Any typographical or grammatical errors should be corrected at revision, so please note any specific errors here.
Reviewer #3: No

Response to reviewers of PLOS ONE
Resubmitted on 20^th^ November 2023 

Following items have been included in the resubmission of the revised manuscript:
•	Rebuttal letter responding to the points raised by the academic editor and reviewers 
•	Revised Manuscript with Track Changes
•	Manuscript 
Authors Response: We appreciate your kind consideration to review our manuscript. We take note of all the comments that you have provided to us. We have responded to all the comments and references are made to the copy of manuscript with track changes. We have responded to the comments on point by point basis as below.
6. Review Comments to the Author

Please use the space provided to explain your answers to the questions above. You may also include additional comments for the author, including concerns about dual publication, research ethics, or publication ethics. (Please upload your review as an attachment if it exceeds 20,000 characters)
Reviewer #3: This revised version of the paper is much improved, however there are numerous grammatical and punctuation errors that need to be addressed. This paper would benefit from a close and thorough copy edit.

Comment: Line 97: injectables?
Response: This has been corrected as injection, see line 97
Comment: Line 104: “Women and girls are vulnerable because they need special care, support, and protection” ?? or “they are vulnerable and they need”… why do they need “special care, support, and protection” ?
Response: Women and girls are vulnerable, and they need care, support and protection… see line 104- 106.
Comment: Line 106: “makes them more susceptible” to what?
Response: susceptible to unintended pregnancies, see lone 106
Comment: Lines 106-107: check punctuation
Response: This has been corrected, see lines 107-109
Comment: Line 125: using qualitative methods (should be methods not method)
Response: This has been changed to methods, see line 118 and 120
Comment: Line 127: same comment re. methods
Response: This has also been addressed, methods, see lines 120
Comment: Line 129: describes
Response: Changed as describes and not described, see line 124
Comment: Lines 200-201: check punctuation – incomplete sentences
Response: This has been addressed and sentence revised, see lines 195 – 197
Comment: Lines 204-205: shouldn't it be “South Sudanese refugee women”
Response: This has been addressed in the entire document and participants referred to South Sudanese refugee women
Comment: Line 213: reached through FGDs? Makes it sound like this was the recruitment modality. Participated in?
Response: This sentence has been revised, see lines 207 - 208
Comment: Line 234: revise sentence - information on contraception is lacking
Response:  The sentence has been restructured, see lines 228- 230
Comment: Line 267: “that personal decision” – which one? Sentence is unclear
Response: Sentence has been revised, see lines 262 – 266
Comment: Line 356: ovaries plural
Response: This has been changed in the revised manuscript, see lines 355
Comment: Line 362: comma unnecessary
Response: This has been revised, see line 396 to 399
Comment: Line 409: sentence incomplete
Response: the sentence has been revised
Comment: Line 431: findings plural
Response: has been corrected
Comment: Lines 444-445: sentence needs revision
Response: Sentence revised, see lines 441 - 443
Comment: Line 510: findings plural 
Response: changed accordingly throughout the manuscript

Comment: Line 522: push on effect? Do you mean knock-on effect? Perhaps better to use more scientific /academic language – e.g. subsequent effect
Response: This change has been effected as subsequent effect, see lie 542
Comment: Line 527: deep rooted
Response: The spelling has been corrected, see line 546
Comment: Line 549: sentence needs revision – grammar incorrect
Response: Sentence has been revised
Comment: Line 596: unfriendly policy framework? Hostile? What do you mean by unfriendly?
Response: The word unfriendly has been changed to hostile, see line 616
Comment: Line 626: dynamics were (dynamics are plural – check grammar)
Response: the word dynamics have been used throughout the document in plural, see line 646
Comment: Lines 631-632: check grammar and punctuation – sentence needs revision – comma is not in the right place
Response: Sentence has been revised in the manuscript 
Comment: Line 634: sentence incomplete
Response: Sentence has been revised in the manuscript 

7. PLOS authors have the option to publish the peer review history of their article (what does this mean?). If published, this will include your full peer review and any attached files.


If you choose “no”, your identity will remain anonymous but your review may still be made public.


Do you want your identity to be public for this peer review? For information about this choice, including consent withdrawal, please see our Privacy Policy.
Reviewer #3: No


[NOTE: If reviewer comments were submitted as an attachment file, they will be attached to this email and accessible via the submission site. Please log into your account, locate the manuscript record, and check for the action link "View Attachments". If this link does not appear, there are no attachment files.]

While revising your submission, please upload your figure files to the Preflight Analysis and Conversion Engine (PACE) digital diagnostic tool, https://pacev2.apexcovantage.com/. PACE helps ensure that figures meet PLOS requirements. To use PACE, you must first register as a user. Registration is free. Then, login and navigate to the UPLOAD tab, where you will find detailed instructions on how to use the tool. If you encounter any issues or have any questions when using PACE, please email PLOS at figures@plos.org. Please note that Supporting Information files do not need this step.


In compliance with data protection regulations, you may request that we remove your personal registration details at any time. (Remove my information/details). Please contact the publication office if you have any questions.
